# Chasing the Ghost: Hyperinflammation Does Not Cause Sepsis

**DOI:** 10.3389/fphar.2022.910516

**Published:** 2022-06-23

**Authors:** Leland Shapiro, Sias Scherger, Carlos Franco-Paredes, Amal A. Gharamti, David Fraulino, Andrés F. Henao-Martinez

**Affiliations:** ^1^ Division of Infectious Diseases, Rocky Mountain Regional Veterans Affairs Medical Center, Aurora, CO, United States; ^2^ Division of Infectious Diseases, Department of Medicine, University of Colorado Anschutz Medical Campus, Aurora, CO, United States; ^3^ Hospital Infantil de México, Federico Gomez, Mexico City, Mexico; ^4^ Department of Internal Medicine, Yale University, Waterbury, CT, United States

**Keywords:** sepsis, inflammation, cytokine storm, COVID-19, tumor necrosis factor, paradigm, Kuhn

## Abstract

Sepsis is infection sufficient to cause illness in the infected host, and more severe forms of sepsis can result in organ malfunction or death. Severe forms of Coronavirus disease-2019 (COVID-19), or disease following infection with severe acute respiratory syndrome coronavirus 2 (SARS-CoV-2) are examples of sepsis. Following infection, sepsis is thought to result from excessive inflammation generated in the infected host, also referred to as a cytokine storm. Sepsis can result in organ malfunction or death. Since COVID-19 is an example of sepsis, the hyperinflammation concept has influenced scientific investigation and treatment approaches to COVID-19. However, decades of laboratory study and more than 100 clinical trials designed to quell inflammation have failed to reduce sepsis mortality. We examine theoretical support underlying widespread belief that hyperinflammation or cytokine storm causes sepsis. Our analysis shows substantial weakness of the hyperinflammation approach to sepsis that includes conceptual confusion and failure to establish a cause-and-effect relationship between hyperinflammation and sepsis. We conclude that anti-inflammation approaches to sepsis therapy have little chance of future success. Therefore, anti-inflammation approaches to treat COVID-19 are likewise at high risk for failure. We find persistence of the cytokine storm concept in sepsis perplexing. Although treatment approaches based on the hyperinflammation concept of pathogenesis have failed, the concept has shown remarkable resilience and appears to be unfalsifiable. An approach to understanding this resilience is to consider the hyperinflammation or cytokine storm concept an example of a scientific paradigm. Thomas Kuhn developed the idea that paradigms generate rules of investigation that both shape and restrict scientific progress. Intrinsic features of scientific paradigms include resistance to falsification in the face of contradictory data and inability of experimentation to generate alternatives to a failing paradigm. We call for rejection of the concept that hyperinflammation or cytokine storm causes sepsis. Using the hyperinflammation or cytokine storm paradigm to guide COVID-19 treatments is likewise unlikely to provide progress. Resources should be redirected to more promising avenues of investigation and treatment.

## 1 Introduction

Sepsis refers to disease caused by infection that can manifest a spectrum of illness ranging from influenza-like discomfort to organ malfunction or death. The standard of care to treat sepsis includes controlling the infectious source, rapid administration of antimicrobial drugs, and supportive measures like volume infusion, oxygen and ventilatory support and use of vasopressors to augment blood pressure (Evans et al., 2021). However, despite advances in standard care, substantial mortality persists in sepsis with overall fatality of 25% and a 40–50% death rate for septic shock (Cavaillon et al., 2020). There is intense clinical interest in lowering this residual mortality. Investigation designed to accomplish this has focused on targeting the pathogen-triggered host inflammatory response to infection. By blocking inflammation as an adjunct therapy to antimicrobials and supportive care, it is hoped sepsis mortality can be lowered. Rationale for pursuing anti-inflammation adjunct therapies focuses on the notion that severe disease following infection is due to an overexuberant host inflammatory reaction. Since pro-inflammatory cytokines are thought to cause inflammation, infection-induced hyperinflammation is often referred to as a cytokine storm (Fajgenbaum and June 2020). According to the hyperinflammation concept, inflammation is a kind of two-edged sword (Chaudhry et al., 2013). A small or appropriate amount of inflammatory response following infection benefits the host by eliminating infection, whereas excessive inflammatory response to infection is detrimental and can result in organ malfunction or death. Interventions designed to suppress host inflammation as a sepsis treatment date to at least 1954 (Spink and Anderson, 1954; Vincent and Abraham, 2006), and a 2021 overview dates the cytokine storm concept back 116 years (Yongzhi, 2021). We believe the hyperinflammation concept of sepsis pathogenesis is mistaken. Recent world events call for urgent re-evaluation of the hyperinflammation concept due to substantial influence this approach has on clinical and research approaches to Coronavirus disease-2019 (COVID-19), the disease following infection with the severe acute respirology syndrome coronavirus 2 (SARS-CoV-2). GOOGLE entries for “inflammation causes COVID-19” reveals 75,500,000 entries and for PubMed this phrase yields 7,649 entries.

This theoretical report brings to light underappreciated problems with the hyperinflammation concept. According to this concept, pro-inflammatory cytokines are produced by host cells after recognizing microbial components and pro-inflammatory cytokines enter the circulation and spread inflammation systemically. Unregulated excessive production of these cytokines is assumed to cause sepsis as an undesired consequence of inflammation run amok. On close inspection, however, we uncover defects with this concept. Two defects we call attention to are conceptual confusion and failure to establish a cause-and-effect relationship between cytokine storm and sepsis. Conceptual confusion refers to a lack of precise understanding of concepts including inflammation, cytokine storm, and sepsis. These ideas are too vague to be used as optimal guides for research or treatment. Lack of clear understanding what these concepts refer to creates challenges in linking investigation and clinical study to sepsis. For example, no consensus defines which cytokines are pro-inflammatory and this complicates interpretation of studies associating specific cytokines with sepsis. These associations may represent true causes of sepsis, non-causal effects of sepsis, or unrelated chance associations with sepsis. A specific understanding of which cytokines are in fact pro-inflammatory would assist interpretation of studies associating specific cytokines with sepsis. Similarly, lack of adequate concepts of inflammation and cytokine storm complicate understanding of sepsis since sepsis pathogenesis depends on these background concepts. An overview of the concepts inflammation, cytokine storm, and sepsis indicates confusion that we believe weakens the relevance of research results to sepsis. We also assessed evidence supporting a cause-effect relationship between cytokine storm and sepsis. Not only is rationale supporting existence of this relationship weak, but available data suggest such a relationship cannot exist.

Finally, we are struck by resistance of the cytokine storm concept of sepsis to yield to falsifying clinical observation. There has been repeated and near exceptionless failure of studies designed to suppress hyperinflammation to treat sepsis. This record has generated little introspection about the viability of the hyperinflammation concept. Cytokine storm appears to be unfalsifiable. Substantial efforts to understand and treat COVID-19 as a manifestation of cytokine storm underscores continued dominance of this concept and establishes it is alive and flourishing in 2022. We believe the remarkable resilience of cytokine storm needs explanation, and we attempted to understand reasons for persistence. The strategy we chose was to consider the hyperinflammation concept of sepsis as a scientific paradigm as described by Kuhn (1962). This strategy not only explains persistence of the cytokine storm concept, but also suggests reasons the concept will likely continue without abandonment in the future. Finally, we propose a way forward that has potential to solve conceptual and practical problems with sepsis.

## 2 Conceptual Confusion

We believe the concept that hyperinflammation is the cause of sepsis is the dominant account of this disease. We therefore assessed pivotal notions associated with this account.

### 2.1 Inflammation

This most central concept in the current approach to sepsis pathogenesis has no established definition and disarray in the sepsis literature has ensued. Discussions of inflammation still emphasize the bedside descriptive characteristics of rubor (redness), tumor (swelling), calor (heat), and dolor (pain). This description is attributed to Celsus in the first century AD. In the 19^th^ century Virchow added loss of function or functio laesa to this list. While much factual knowledge was accrued regarding clinical (bedside), histological, and molecular components of the inflammatory response, there remains no established unifying concept relating these components. No concise definition of inflammation has been established (Kushner, 1998; Groopman, 2015; Antonelli and Kushner, 2017). In addition to uncovering what regularities lie at the heart of inflammation, there is need to unify notions of local and systemic inflammation so they can be understood as manifestations of a single process. This point is crucial since the definition of sepsis is predicated on understanding systemic inflammation. Prior to the sepsis 3 definition of sepsis, a systemic inflammatory response syndrome (SIRS) caused by infection was defined as sepsis (Gul et al., 2017). Sepsis 3 characterizes sepsis as a dysregulated immune response to infection with organ malfunction. A dysregulation of immune response is unhelpful as a description of how the disease is caused, and there is no principled way to understand how this characterization differs from overactive systemic inflammation. Regardless, sepsis 3 focuses on infection-associated organ pathology with de-emphasis on underlying cause.

Confusion about inflammation has been noted and reviewed by Groopman (2015), in the FASEB Journal by Weissmann (2010) and later by Antonelli and Kushner (2017). There seems to be little agreement on what comprises inflammation other than association with innate immune antimicrobial activity, where innate immunity refers to host antipathogen function that does not require prior exposure to pathogen. Inflammation is thought to relate to innate immunity, and it is often implied some components of innate immunity are separate from inflammation. There is need for a definition of inflammation that captures its role in innate immunity, relates local and systemic inflammation, and unifies manifestations at the bedside, in histological specimens, and at the molecular level. A desirable quality of a novel understanding and definition would be a description that permits precise demarcation between inflammation and non-inflammatory phenomena like acquired immunity and autoimmunity. Our group is devising a novel definition of inflammation that we believe can accomplish many of these goals (manuscript in preparation).

### 2.2 Cytokine Storm

This is a nebulous term with an uncertain definition. Use of “cytokine storm” dates to at least 1993 in a description of graft vs host disease (Ferrara et al., 1993). A recent review summarized current thinking on this concept and includes discussion of the related cytokine release syndrome (Fajgenbaum and June 2020). Cytokine storm refers to overexuberant cytokine synthesis in response to a pathogen or other stimulus that causes adverse clinical manifestations that can include constitutional symptoms (including fever), organ malfunction, coagulopathy, epithelial barrier incompetence, or death (Fajgenbaum and June 2020). An explosion of cytokine research has uncovered over 100 genes coding cytokine-like activities, including at least 52 chemokines and 43 interleukins (Dinarello, 2007; Turner et al., 2014). Some cytokines are described as possessing inflammatory activities whereas others are described as possessing anti-inflammatory function. This has caused further confusion since there is no adequate definition of inflammation that permits a principled classification of cytokines as pro-inflammatory or anti-inflammatory. Recent technological advances have produced multiplex assay platforms that can determine numerous cytokines and other molecules in single biological (often blood) samples rapidly and at low cost. These technological achievements combined with absence of an applicable definition of inflammation has resulted in a chaotic literature. Identical molecules are sometimes described as pro-inflammatory or anti-inflammatory in different reports. A prototype example is interleukin (IL)-6, which is described as pro-inflammatory in reports of its role in COVID-19 (see below) despite reports showing IL-6 inhibits induction of the prototype pro-inflammatory cytokine tumor necrosis factor alpha (TNF) (Aderka et al., 1989; Schindler et al., 1990; Dinarello, 1991a). A sample of publications reporting circulating cytokine levels in COVID-19 shows measurement of circulating cytokines numbering 27 (Huang et al., 2020), 48 (Chi et al., 2020), 48 (Tjan et al., 2021), 35 (Mudd et al., 2020), and 53 (Herr et al., 2021). The GOOGLE entry “Cytokine levels in COVID-19” reveals 11, 400,000 entries and for PubMed 2,885 publications. Some studies identify molecules as cytokines that are not commonly considered cytokines like complement, tumor markers, ferritin, and CRP or procalcitonin. Perhaps no two reports describing cytokine levels in COVID-19 measure the same menu of cytokines, and no two reports disclose the same ensemble of significantly elevated cytokines. In the absence of an accepted definition of inflammation that precisely defines which cytokines are pro-inflammatory, it is understandable there is little agreement which cytokines are pro-inflammatory. Multiplex assays used to measure cytokines or other molecules in blood that show significant differences between COVID-19 patients and a comparator group (often healthy controls or COVID-19 patients with different severities) generates more opacity than clarity if used to uncover pathogenesis or devise therapeutic targets. Confusion associated with the concept of cytokine storm motivated us to devise a novel characterization of inflammation that includes structured criteria defining cytokines as pro-inflammatory or anti-inflammatory (manuscript in preparation).

### 2.3 Sepsis

Sepsis describes significant clinical disease following infection of any kind. The global significance of this disease is underscored by a report revealing 48.8 million sepsis cases and 11.0 million sepsis deaths that accounted for 18.7% of global deaths in 2017 (Rudd et al., 2020). In 2017 the World Health Organization designated sepsis a global health priority (Reinhart et al., 2017). The concept of pathogenesis that has dominated thinking and clinical study in sepsis is the role of hyperinflammation as cause (Dinarello, 1991b; Cross et al., 1993; Hotchkiss and Karl, 2003; Martin et al., 2003; Vincent and Abraham, 2006; Rittirsch et al., 2007; Marshall, 2014; Tsirigotis et al., 2016; Cavaillon et al., 2020; De Stefano et al., 2020; Jarczak et al., 2021). The focus on inflammation is clear when examining accepted criteria or definitions of sepsis (Mayr et al., 2014; Gul et al., 2017). The 1991 sepsis 1 definition formulated by the American College of Chest Physicians and Society of Critical Care Medicine (SCCM) defined sepsis as infection-associated systemic inflammatory response syndrome or SIRS. The SIRS criteria required 2 or more clinical findings from a list that included: fever or hypothermia, tachycardia, tachypnea or hypocapnia, and leukocytosis or leukopenia. The sepsis 1 definition suffered low specificity since non-infectious conditions could satisfy SIRS criteria, including post-surgery illness, drug effects, pancreatitis, trauma, burns, ischemia-reperfusion, and others. The 2001 sepsis 2 definition characterized sepsis as infection associated with expanded menus of general, inflammatory, hemodynamic, organ malfunction, and tissue perfusion anomalies. However, the diagnostic criteria were unaltered between sepsis 1 and sepsis 2. The sepsis 3 definition published 2016 (task force derived from the Society of Critical Care Medicine and the European Society of Intensive Care Medicine) eliminated SIRS criteria due to low sensitivity and specificity (Singer et al., 2016). Sepsis 3 focused on established or presumed infection and associated organ malfunction defined as increase in 2 or more points in the sequential organ failure assessment (SOFA) score. The sepsis 3 criteria marked a conceptual break with sepsis 1 and sepsis 2 definitions since evidence of underlying systemic inflammation was eliminated. Inadequate sensitivity and specificity motivated elimination of systemic inflammation criteria from sepsis 3. However, sepsis 3 broadly describes sepsis as a dysregulated immune response to infection. While we do not believe this phrase has a clear meaning, it suggests sepsis 3 retains reference to underlying excessive inflammation. This characterization permeates all three sepsis definitions. However, if sepsis is in fact not a disease caused by inflammation, attempts to link organ malfunction or death to inflammation will fail. We believe sepsis is not caused by any kind of hyperinflammation (Section 3). In retrospect, sepsis definitions have included paradoxical criteria including either elevated or reduced temperature and either increased or decreased white blood cell counts. Although inclusion of these peculiar criteria in sepsis 1 and sepsis 2 definitions were invoked to capture patients suffering disease due to infection-induced hyperinflammation, they call into question the conceptual underpinnings of sepsis.

### 2.4 Two-Edged Sword

This is an analogy invoked to describe the role of inflammation in host-pathogen interaction (Chaudhry et al., 2013). Properly regulated inflammation generated at appropriate levels and at the appropriate time benefits the host in eliminating pathogens. However, if inflammation is excessive or somehow dysregulated, host-derived inflammation becomes detrimental and can lead to organ malfunction or death. Conceptual difficulties are evident. If the inflammatory response is characterized as dysregulated (as in the sepsis 3 definition), little is gained other than asserting something has gone wrong. How a balance between pro-inflammatory and anti-inflammatory molecules relates to sepsis is undefined. A related defect in the two-edged sword analogy concerns quantification of inflammation mediators. The sword analogy clearly implies increased counterproductive inflammation exists in sepsis patients. However, no one has characterized which cytokines (or other molecules) participate as mediators of inflammation. Moreover, mediator concentrations that separate adaptive beneficial amounts from excessive detrimental levels are not specified in the literature. Poor characterization of identity and concentrations of cytokines that cause sepsis is a substantial conceptual defect. In response to this lacuna in the literature, our group has conducted a systematic review and meta-analysis that reports concentration of pro-inflammatory cytokines in the circulation of sepsis patients (manuscript in review) (Gharamti et al., 2021).

## 3 Cause Analysis; Hyperinflammation Does Not Cause Sepsis

The concept host inflammation causes organ damage or death following infection appears to date to two discoveries (Marshall, 2014). One was characterization of endogenous pyrogens or host-derived fever-inducing molecules that included the cytokine TNF (Dinarello, 1999). Since bedside observation associated fever with inflammation, characterization of endogenous pyrogen molecules suggested inflammation originated from host substances produced during infection. Attention focused on a host role in clinical manifestations of infection. The second discovery was characterization of special mice that resisted lethality in endotoxin models of sepsis (Sultzer, 1972; Lehmann et al., 1987). Injection of endotoxin surface components of gram-negative bacteria induced inflammation and physiological responses that resembled sepsis (Raetz and Whitfield, 2002; Rittirsch et al., 2007). Parenteral endotoxin administered at levels that killed normal mice showed no lethality in special endotoxin resistant mice. Therefore, mice that did not succumb to endotoxin infusion suggested host factors of some sort were interposed between endotoxin injection and sepsis mortality. Elegant experiments showed transfer of bone marrow cells from normal endotoxin-sensitive mice into special endotoxin-resistant mice converted resistant mice into endotoxin sensitive mice (Michalek et al., 1980). Therefore, some characteristic of white blood cells from special endotoxin resistant mice differed from normal endotoxin-sensitive mice. Since endotoxin injection was thought to mimic sepsis, it was believed components of host white blood cells were somehow mediating sepsis lethality. Here again, attention was called to a host role in infection. Moreover, characteristics of host white blood cells seemed to account for host lethality in the endotoxin sepsis model. It appears a role for microbial pathogens as direct causes of sepsis was concomitantly de-emphasized. A concept seemed to emerge that pathogens did not directly kill sepsis patients, but the response to pathogens caused sepsis patients to kill themselves. It was later discovered these special endotoxin unresponsive mice harbored a loss of function mutation in the pattern recognition receptor that identifies endotoxin (Qureshi et al., 1999). A link between the endotoxin sepsis model and white blood cell biology was demonstrated when specific blockade of TNF lowered mortality in an endotoxin mouse model of sepsis (Beutler et al., 1985). Since white blood cells are considered a primary source of TNF during sepsis, the combination of observations described above suggested infections supplied an endotoxin source that induced white blood cells to produce excessive TNF that in turn caused sepsis organ malfunction and mortality (Bradley, 2008). Importantly, it appeared blockade of the host cytokine TNF induced by bacterial endotoxin could lower mortality in sepsis.

TNF does not directly damage tissues, but TNF initiates biological activities that can culminate in tissue damage. Proposed mechanisms invoked to link TNF excess to toxicity in sepsis include polymorphonuclear neutrophil (PMN) chemotaxis directed into tissues (Smart and Casale, 1994; Vieira et al., 2009). Tumor necrosis factor can also activate PMNs. Activated PMNs can secrete reactive oxygen species, neutrophil elastase, and neutrophil extracellular traps, which may individually or in concert damage tissues (Bajaj et al., 1992; Ferrante, 1992; Keshari et al., 2012). Other mechanisms invoked to explain TNF-mediated tissue damage include activation of complement or coagulation cascades (van der Poll et al., 1990; Page et al., 2018; Gao et al., 2019). Sepsis-associated complement deposition on host cells can in principle cause cell damage, and coagulation activation may induce disseminated thromboses that may result in tissue ischemia.

### 3.1 Evidence That Inflammation Causes Sepsis

To evaluate the claim hyperinflammation or cytokine storm causes sepsis, we focus on the strength of evidence for TNF as cause for five reasons. First, we believe sound theoretical reasons support TNF as a pivotal cause of inflammation (manuscript in preparation). Tumor necrosis factor has been labeled the “master regulator” of inflammatory cytokine production (Parameswaran and Patial, 2010), and referred to as “the prime mediator of the inflammatory response seen in sepsis and septic shock” (Spooner et al., 1992). Second, TNF injection into animals results in altered physiology thought to mimic sepsis (Tracey et al., 1986; Bauss et al., 1987; Spooner et al., 1992), Third, neutralization of TNF activity has been widely studied as a therapy in animal sepsis models (Beutler et al., 1985; Tracey et al., 1986; Tracey et al., 1987; Mathison et al., 1988; Fong et al., 1989; Bajaj et al., 1992; Mohler et al., 1993; Mullen et al., 1994; Remick et al., 1995; Beutler et al., 2008; Bojalil et al., 2013). Fourth, the history of anti-inflammation strategies employed to treat human sepsis has emphasized specific inhibition of TNF as a sepsis treatment. (Table 1). Fifth, more studies are available that can be used to assess sepsis causality for TNF than for other molecules. These considerations support a focus on TNF as the prototype cytokine thought to cause the hyperinflammation of sepsis (Bradley, 2008). We believe three criteria can be used to conclude excessive inflammation causes sepsis. These include 1) showing pro-inflammatory cytokines are necessary for sepsis, 2) showing pro-inflammatory cytokines are sufficient for sepsis, and 3) showing pro-inflammatory cytokines satisfy an interventionist account of causality.

**TABLE 1:** Clinical studies evaluating anti-inflammation strategies to treat sepsis.

Intervention	References	Citation in references	Notes
Corticosteroids	1. 1984/Sprung CL et al., MEJM, vol 311(18), pages 1137,1143, 1984	Sprung et al. (1984)	
	2. 1987/Bone RC *et al*, NEJM, vol 317(11), pages 653–658, 1987	Bone et al. (1987)	
	3. 1987/The Veterans Administration Systemic Sepsis Cooperative Study Group, NEJM, vol 317(11), pages 659–665, 1987	Veterans Administration Systemic Sepsis Cooperative Study Group, (1987)	
	4. 2002/Annane D *et al*, JAMA, vol vol 288(7), pages 863–871, 2002	Annane et al. (2002)	
	5. 2007/Cicarelli DD *et al*, Sao Paulo Medical Journal, vol 25(4), pages 237–241, 2007	Cicarelli et al. (2007)	
	6. 2008/Sprung CL *et al*, NEJM, vol 358(2), pages 111–124, 2008	Sprung et al. (2008)	
	7. 2016/Keh D *et al*, JAMA, vol 316(17), pages 1775–1785, 2016	Keh et al. (2016)	
	8. 2016/Gordon AC *et al*, JAMA vol 316(5), pages 509–518, 2016	Gordon et al. (2016)	
	9. 2018/Venkatesh B *et al*, NEJM, vol 378(9), pages 797–808, 2018	Venkatesh et al. (2018)	
Aspirin	1. 2016/Kor DJ *et al*, JAMA, 15 May 2016 (E-pub)	Kor et al. (2016)	Randomized controlled study of aspirin prophylaxis to avert acute respiratory distress syndrome; about 77% of patients with suspected sepsis on enrollment-no mortality. difference with aspirin.
Acetaminophen	1. 2015/Young P *et al*, NEJM, vol 373(23), pages 2215–2224, 2015	Young et al. (2015)	
Ibuprofen	1. 1991/Haupt MT *et al*, Critical Care Medicine, vol 19(11), pages 1339–1347, 1991	Haupt et al. (1991)	
	2. 1997/Bernard GR *et al*, NEJM, vol 336(13), pages 912–918, 1997	Bernard et al. (1997)	
Physical Cooling	1. 2013/Yang Y-L *et al*, Chinese Medical Journal, vol 126(10), pages 1809–1813, 2013	Yang et al. (2013)	Water-flow cooling blankets. Increased mortality in experimental group (statistically significant).
Neutrophil Elastase Inhibitor	1. Zeiher BG *et al*, Critical Care Medicine, vol 32(8), pages 1695–1702, 2004	Zeiher et al. (2004)	Small molecule inhibitor in patients with acute lung injury, of whom 58.5% were caused by infection. Subgroup with pulmonary infection showed no mortality benefit.
Phospholipase A2 inhibition	1. 2003/Abraham E *et al*, Critical Care Medicine, vol 31(3), pages 718–728, 2003	Abraham et al. (2003a)	
Anti-endotoxin antibodies	1. 1991/Ziegler EJ *et al*, NEJM, vol 324(7), pages 429–438, 1991	Ziegler et al. (1991)	
	2. 1991/Greenman RL *et al*, JAMA, vol 266(8), pages 1097–1102, 1991	Greenman et al. (1991)	
	3. 1994/McCloskey RV *et al*, Annals of Internal Medicine, vol 121(1), pages 1–5, 1994	McCloskey et al. (1994)	
	4. 1999/Derkx B *et al*, Clinical Infectious Diseases, vol 28, pages 770–777, 1999	Derkx et al. (1999)	
TLR4 antagonist (synthetic)	1. 2010/Rice TW *et al*, Critical Care Medicine, vol 38(8), pages 1–10, 2010	Rice et al. (2010)	
	2. 2013/Opal SM *et al*, JAMA, vol 309(11), pages 1154–1162, 2013	Opal et al. (2013)	
Endotoxin hemofiltration	1. 2014/Iwagami M *et al*, Critical Care Medicine, vol 42(5), pages 1187–1193, 2014	Iwagami et al. (2014)	
Polymyxin B hemoperfusion	1. 2018/Dellinger RP *et al*, JAMA, vol 320(14), pages 1455–1463, 2018	Dellinger et al. (2018)	
	2. 2015/Payen DM *et al*, Intensive Care Medicine, vol 41(6), pages 975–984, 2015	Payen et al. (2015)	
	3. 2009/Cruz DN *et al*, JAMA, vol 301(23), pages 2445–2452, 2009	Cruz et al. (2009)	
Bactericidal/Permeability Increasing Protein	1. 2000/Levin M *et al*, The Lancet, vol 356(9234), pages 961–967, 2000	Levin et al. (2000)	Recombinant protein.
Continuous veno-venous hemofiltration (low volume)	1. 2009/Payen D *et al*, Critical Care medicine, vol 37(3), pages 803–810, 2009	Payen et al. (2009)	
Continuous veno-venous hemofiltration (high volume)	1. 2013/Joannes-Boyau O *et al*, Intensive Care Medicine, vol 39(9), pages 1535–1546, 2013	Joannes-Boyau et al. (2013)	Meta-analysis of randomized controlled studies in N = 5 studies (N = 241 subjects) showing no differences between high volume hemofiltration vs low volume hemofiltration in Sepsis. Since above study showed no beneficial effect of low volume hemofiltration it is implied high-volume hemofiltration is likewise ineffective vs sepsis.
	2. 2020/Yin F *et al*, Annals of Translational Medicine, vol 8(7), pages 1–10, 2020	Yin et al. (2020)	
Plasma exchange	1. 2014/Rimmer E *et al*, Critical Care, vol 18(6), pages 1–8, 2014	Rimmer et al. (2014)	Systematic review and meta-analysis identified 4 randomized controlled trials in pateints with sepsis or septic shock. Overall, no benefit but found benefit if analysis restricted to adult patients (N = 128) but not in children (N = 66).
CytoSorb^Ⓡ^ Extracorporeal Cytokine Hemadsorption	1. 2017/Schädler D *et al*, Plos One, vol 12(10), pages 1–18, 2017	Schadler et al. (2017)	Randomized controlled open-label study (N = 100, mortality = secondary outcome).
Coupled plasma filtration adsorption	1. 2014/Livigni S *et al*, BMJ Open, vol January 8, 4(1), pages 1–10, 2014	Livigni et al. (2014)	
Phospholipid emulsion	1. 2009/Dellinger RP *et al*, Critical Care Medicine, vol 37(11), pages 2029–2038, 2009	Dellinger et al. (2009)	
Nitric oxide inhibition	1. 2004/Lopez A *et al*, Critical Care Medicine, vol 32(1), pages 21–30, 2004	Lopez et al. (2004)	Increased mortality in experimental group (statistically significant).
Bradykinin antagonist (synthetic)	1. 1997/Fein AM *et al*, JAMA, vol 277(6), pages 482–487, 1997	Fein et al. (1997)	
Antithrombin-3 (natural)	1. 2001/Warren BL *et al*, JAMA, vol 286(15), pages 1868–1878, 2001	Warren et al. (2001)	
	2. 2013/Gando S *et al*, Critical Care, vol 17(6) R297, pages 1–10, 2013	Gando et al. (2013)	
Tissue factor pathway inhibitor (recombinant)	1. 2001/Abraham E *et al*, Critical Care Medicine, vol 29(11), pages 2081–2088, 2001	Abraham et al. (2001)	
	2. 2003/Abraham E *et al*, JAMA, vol 290(2), pages 238–247, 2003	Abraham et al. (2003b)	
Soluble human thrombomodulin (human recombinant)	1. 2013/Vincent J-L *et al*, Critical Care Medicine, vol 41(9), pages 2069–2079, 2013	Vincent et al. (2013)	Review and meta-analysis of drug approved for DIC therapy in Japan; included 3 randomized controlled trials (N = 838) showing no 28–30 days mortality benefit in adult DIC patients with sepsis or severe sepsis.
	2. 2014/Yamakawa K *et al*, Journal of Thrombosis and Hemostasis, vol 13 (4), pages 508–519, 2014	Yamakawa et al. (2015)	
	3. 2019/Vincent J-L *et al*, JAMA, vol 321(20), pages 1993–2002, 2019	Vincent et al. (2019)	
Heparin (Heparin possesses anti-inflammatory properties)	1. 2007/Levi M *et al*, American Journal of Respiratory and Critical Care Medicine, vol 176, pages 483–490, 2007	Levi et al. (2007)	Randomized assignment to heparin in patients given activated protein C. Systematic review and meta-analysis (9 randomized controlled trials with 2,637 patients).
	2. 2009/Jaimes F *et al*, Critical Care Medicine, vol 37(4), pages 1185–1196), 2009	Jaimes et al. (2009)	
	3. 2015/Zarychanski R *et al*, Critical Care Medicine, vol 43(3), pages 511–518, 2015	Zarychanski et al. (2015)	
Activated Protein C (recombinant)	1. 2001/Bernard GR *et al*, NEJM, vol 344(10), pages 699–709, 2001	Bernard et al. (2001)	Positive study- could not be replicated.
	2. 2005/Abraham E *et al*, NEJM, vol 353(13), pages 1332–1341, 2005	Abraham et al. (2005)	
	3. 2007/Nadel *et al*, LANCET, vol 369, pages 836–843, 2007	Nadel et al. (2007)	
	4. 2012/Ranieri VM *et al*, NEJM, vol 366(2), pages 2055–2064, 2012	Ranieri et al. (2012)	
IL-1 receptor antagonist (recombinant)	1. 1994/Fisher CJ *et al*, Critical Care Medicine, vol 22(1), pages 12–21, 1994	Fisher et al. (1994b)	Remarkable open label randomized placebo-controlled study showing dose-response mortality reduction.
	2. 1994/Fisher CJ *et al*, JAMA, vol 271(23), pages 1836–1843, 1994	Fisher et al. (1994a)	
	3. 1997/Opal SM *et al*, Critical Care Medicine, vol 25(7), pages 1115–1124, 1997	Opal et al. (1997)	
TNF Antagonists	1. 1993/Fisher CJ *et al*, Critical Care Medicine, vol 21(3), pages 318–327, 1993	Fisher et al. (1993)
	2. 1995/Abraham E *et al*, JAMA, vol 273(12), pages 934–941, 1995	Abraham et al. (1995)
	3. 1996/Fisher CJ *et al*, NEJM, vol 334(26), pages 1697–1702, 1996	Fisher et al. (1996)	Used etanercept (Enbrel^®^). Increased mortality in experimental group (statistically significant).
	4. 1996/Cohen J and Carlet J, Critical Care Medicine, vol 24(9), pages 1431–1440, 1996	Cohen and Carlet, (1996)	
	5. 1996/Reinhart K *et al*, Critical Care Medicine, vol 24(5), pages 733–742, 1996	Reinhart et al. (1996)	
	6. 1998/Abraham E, Lancet, vol 351(9107), pages 929–933, 1998	Abraham et al. (1998)
	7. 2001/Reinhart K *et al*, Critical Care Medicine, vol 29(4), pages 765–769, 2001	Reinhart et al. (2001)
	8. 2004/Panacek EA *et al*, Critical Care Medicine, vol 32(11), pages 2173–2182, 2004	Panacek et al. (2004)	Reported as positive study, but restricted to subgroup with IL-6>1,000 and using post-study logistic regression to balance data (both elements established prospectively).
	9. 2006/Rice TW *et al*, Critical Care Medicine, vol 34(9), pages 2271–2281, 2006	Rice et al. (2006)	
Platelet Activating factor receptor antagonist	1. 1994/Dhainaut JF *et al*, Critical Care Medicine, vol 22(11), pages 1720–1728, 1994	Dhainaut et al. (1994)	
	2. 1998/Dhainaut JF *et al*, Critical Care Medicine, vol 26(12)), pages 1963–1971, 1998	Dhainaut et al. (1998)	
	3. 2000/Suputtamongkol Y *et al*, Antimicrobial Agents and Chemotherapy, vol 44(3), pages 693–696, 2000	Suputtamongkol et al. (2000)	
	4. 2000/Vincent JL *et al*, Critical Care Medicine, vol 28(3), pages 638–642, 2000	Vincent et al. (2000)	
	5. 2000/Poeze M *et al*, Shock, vol 14(4), pages 421–428, 2000	Poeze et al. (2000)	
	6. 2004/Opal S *et al*, Critical Care Medicine, vol 32(2), pages 332–341, 2004	Opal et al. (2004)	
Statins	1. 2013/Kruger P *et al*, American Journal of Respiratory and Critical Care Medicine, Vol 187(7), pages 743–750, 2013	Kruger et al. (2013)	Meta-analysis (4 randomized controlled studies, 1818 adult patients with severe sepsis). Systematic review and meta-analysis (7 randomized controlled studies, 1720 adult patients with sepsis). Systematic review of 8 randomized controlled studies (N = 2,275 adults with sepsis).
	2. 2014/McAuley DF *et al*, New England Journal of Medline, vol 371(18), pages 1695–1703, 2014	McAuley et al. (2014)	
	3. 2015/Thomas G *et al*, Minerva Anestesiologica, vol 81(8), pages 921–930, 2015	Thomas et al. (2015)	
	4. 2015/Deshpande, A. *et al*, American Journal of Medicine, vol 128, pages 410–417, 2015	Deshpande et al. (2015)	
	5. 2016/Quinn M et al., Indian Journal of Critical Care Medicine, vol 20(9), pages 534–541, 2016	Quinn et al. (2016)	
Vitamin C + Hydrocortisone + Thiamine	1. 2016/Marik PE *et al*, Chest, vol 151(6), pages 1229–1238, 2016.	Marik et al. (2017)	Substantial reduced mortality in non-contemporaneous controls (hospital mortality = 8.5% vs 40.4% = 79% relative mortality reduction). Septic shock in adults in prospective, randomized, controlled (hydrocortisone alone) trial that was OPEN LABEL; no effect for outcome of alive and vasopressor-free duration. Subgroups showed no mortality benefit for combination therapy at days 28, 90, or in ICU (in fact, mortality slightly increased for intervention for all mortality assessments). Another study with non-contemporaneous controls propensity-score matched showed no mortality benefit (mortality in ICU or 28 days or 60 days). No change in SOFA score over first 3 days. Intervention showed reduced length of stay in ICU or in hospital.
	2. 2020/Fujii T *et al*, JAMA vol 324(5), pages 423–431, 2020	Fujii et al. (2020)	
	3. 2020/Mitchell AB *et al*, American Journal of Medicine, vol 133(5), pages 635–638, 2020	Mitchell et al. (2020)	

### 3.2 Necessary Condition

Evidence that TNF is necessary for sepsis amounts to showing TNF is elevated in sepsis patients compared to healthy controls. Remarkably, no report comprehensively quantifies circulating concentrations of TNF in healthy persons and in patients with sepsis. Therefore, we conducted a systematic review and meta-analysis that addressees this lacuna in the literature and submitted our manuscript that is currently being subjected to peer review. This manuscript is based on a prospectively published research plan (Gharamti et al., 2021), and the manuscript is available in an open-access repository (PROSPERO registration number CRD42020179800) and see https://www.medrxiv.org/content/10.1101/2021.12.13.21267720v1.full.pdf. We found healthy persons have approximately 5.5 pg/ml TNF in the circulation, and in sepsis patients TNF concentration increases to approximately 60 pg/ml. Therefore, the case for a necessary increase in cytokines in sepsis is reasonable with levels approximately 10-fold elevated compared to healthy persons. This criterion of causality establishes a consistent relationship between sepsis and increased pro-inflammatory cytokines.

### 3.3 Sufficient Condition

To show TNF is sufficient for sepsis, TNF should be capable of producing sepsis. The best available data derive from studies reporting injecting humans with TNF and observing for sepsis manifestations. Recombinant TNF has been injected intravenously into volunteers, usually as attempts to treat cancer (Chapman et al., 1987; Starnes et al., 1988). In general, injection with recombinant TNF can produce fever, tachycardia, and occasional fluid-responsive hypotension at high injection doses; no severe organ dysfunction or death has been reported. However, circulating TNF concentrations following recombinant TNF intravenous infusions were several orders of magnitude larger than amounts in sepsis patients. Serum or plasma TNF levels in human infusion studies were calculated based on dose or directly measured. We calculated TNF serum levels of approximately 100,000 pg/ml—200,000 pg/ml in studies that did not measure TNF, and studies that measured TNF found about 10,000—80,000 pg/ml in circulation (Blick et al., 1987; Chapman et al., 1987; Starnes et al., 1988). We believe available reports cannot support the criterion that pro-inflammatory cytokines are sufficient for sepsis, since injected amounts of TNF vastly exceeded levels that we determined to be present in natural sepsis (approximately 60 pg/ml). Even spectacularly large amounts of infused TNF fail to produce the severe detrimental effects (including death) of natural sepsis. It seems highly unlikely that infusion of TNF at amounts designed to produce circulating levels corresponding to those in natural sepsis will result in clinical effects exceeding influenza-like illness.

### 3.4 Interventionist Condition

If deliberately altering a putative cause by changing its magnitude or eliminating its presence entirely results in consistent alterations in the putative effect, then a cause-effect relationship is detected. Intuitively, if you “wiggle” the cause, you should “jiggle” the effect. This comprises the interventionist or manipulationist account of causality. This interventionist criterion comprises a powerful method for establishing cause-effect relationships and is useful in explaining how bioscience researchers establish causality in the laboratory or in clinical investigation. James Woodward is a champion of this concept which he describes as “counterfactual invariance under intervention” (Woodward, 2003). This is an epistemic or detection concept of causality, meaning it is a rule or guide that permits one to recognize cause and effect relationships when such relationships exist. Discovering that hyperinflammation or cytokine storm is a cause of sepsis would be satisfied by showing deliberate variation (intervention) in cytokines results in alteration in sepsis. Translating this account of causality into experimental design has focused on sepsis diminution (effect) following blockade (manipulation) of cytokines (cause). Sepsis diminution usually comprises reducing sepsis mortality at 28 days after diagnosis. Since an important goal of showing cytokine storm causes sepsis is to benefit patients, choosing mortality as outcome selects the ultimate benefit. Anti-inflammation interventions designed to treat sepsis are intended for use as adjunct therapies in addition to standard of care measures like antimicrobial drugs, fluid administration, pressor support, mechanical ventilation, vasopressors, and other measures (Evans et al., 2021). Unfortunately, all attempts to satisfy the manipulation criterion to show hyperinflammation causes sepsis have failed. No intervention designed to suppress inflammation or cytokine storm has demonstrated proven clinical benefit, where benefit refers to 28–30 days mortality as outcome. Table 1 shows a non-exhaustive collection of clinical studies that have failed to show beneficial effect for inflammation-blocking interventions. We present this table to underscore the many kinds of inflammation-defeating measures that have been tested. The magnitude of this effort demonstrates failure has not been due to lack of enthusiasm for the concept, lack of resources, or lack of imagination designing novel ways to block inflammation. Reviews of the status of immunomodulator therapies tested for sepsis treatments are typified by this 2014 comment in Opal et al. (2014): “Hundreds of millions of dollars have been expended enrolling over 30,000 patients in clinical trials to test and develop new immunomodulating agents, anti-inflammatory agents, and antiendotoxin agents. Yet, not a single agent has convincingly proven to be consistently efficacious in clinical trials. There are no new drugs on the market to show for all this effort.” Marshall wrote in 2014 “There have been more than 100 Phase II and Phase III clinical rials of strategies to modify the systemic inflammatory response by selectively or nonselective targeting its endogenous mediator molecules” (Marshall, 2014). Marik *et al* emphasized and expanded on this observation in 2017: “Over the last 3 decades, more than 100 phase 2 and phase 3 clinical trials have been performed testing various novel pharmacologic agents and therapeutic interventions in an attempt to improve the outcome of patients with severe sepsis and septic shock; all of these efforts ultimately failed to produce a novel pharmacologic agent that improved the outcome of sepsis” (Marik et al., 2017). Clearly, the hyperinflammation or cytokine storm concept has failed the interventionist criterion for sepsis causality.

## 4 Why Hyperinflammation Does Not Cause Sepsis

We do not believe hyperinflammation or cytokine storm is a cause of sepsis. Several lines of evidence support our contention. First, all attempts to block inflammation by numerous approaches have failed to reduce sepsis mortality (Table 1). In fact, we are not aware of any clinical study or case report definitively showing hyperinflammation caused organ malfunction of death. Second, quantification of circulating TNF in natural sepsis revealed concentrations inadequate to cause organ damage or death. In fact, our study determined counterintuitively low TNF levels in sepsis patients https://www.medrxiv.org/content/10.1101/2021.12.13.21267720v1.full.pdf. The circulating TNF levels of approximately 60 pg/ml in sepsis patients is orders of magnitude below concentrations easily tolerated by humans following injection of recombinant TNF (Section 3). Third, concentrations of TNF in sepsis are comparable to levels observed in common non-lethal diseases like rheumatoid arthritis, inflammatory bowel disease, and Streptococcal pharyngitis, casting further doubt that sepsis-associated inflammation causes sepsis (manuscript in preparation).

## 5 COVID-19

Since sepsis entails linkage between infection and clinical illness, COVID-19 patients can satisfy criteria for sepsis, severe sepsis, or septic shock (Lin et al., 2018; Gu et al., 2020). Importantly, since sepsis is presumed to be caused by hyperinflammation, COVID-19 sepsis adopts the view hyperinflammation or cytokine storm causes illness. Coronavirus disease 2019 has called attention of the hyperinflammation/cytokine storm concept like no previous disease. Remarkably, GOOGLE entries for “Inflammation causes COVID-19” numbers 82,800,000. Articles relating cytokine storm to COVID-19 have appeared in at least 8 of the 10 top US newspapers with largest circulations: The Wall Street Journal, USA Today, The New York Times, New York Daily News, The Washington Post, Minneapolis Star Tribune, Chicago Tribune, and Los Angeles times. The hyperinflammation concept has clearly influenced activities of The Coronavirus Treatment Acceleration Program (CTAP) that was created by the United States Food and Drug Administration (FDA) to rapidly assess and promote potential COVID-19 treatments (https://www.fda.gov/drugs/coronavirus-covid-19-drugs/coronavirus-treatment-acceleration-program-CTAP). The CTAP dashboard lists types of treatments under consideration with “safe to proceed investigational new drugs”. As of 10 March 2022, CTAP listed (excluding vaccines) more than 120 immunomodulator (mostly anti-inflammation) therapies, followed by “other” treatments with more than 110, neutralizing antibodies or cell and gene therapies with over 60 each, and antiviral approaches with more than 50 entries. This snapshot reflects the degree to which inflammation suppressing approaches influence research priorities in developing COVID-19 therapies. Anti-inflammation drugs to treat COVID-19 were investigated early during the pandemic. Following rapid conduct of clinical studies to treat COVID-19, accepted cytokine-suppressing treatments that reportedly lower mortality include drugs that block IL-6 biological activity and the immunosuppressive corticosteroid dexamethasone (Agarwal et al., 2020). Focusing on IL-6 inhibition, comprehensive meta-analyses of randomized controlled trials in hospitalized COVID-19 patients reported mortality benefit for the anti-IL-6 receptor antibody tocilizumab (Tharmarajah et al., 2021; WHO Rapid Evidence Appraisal for COVID-19 Therapies (REACT) Working Group, 2021). However, the history of failed immune-suppressing or cytokine-inhibiting treatments to treat sepsis (Table 1) suggests caution in accepting this conclusion. As elaborated above (Section 3), we add theoretical considerations suggesting inflammation is not the cause of sepsis as reason to closely scrutinize claims to the contrary. In fact, at least three reasons suggest rationale for IL-6 inhibitor COVID-19 benefit is suspect. Reason one is discrepant mortality results for IL-6-blocking drugs with similar mechanisms of biological activity. One meta-analysis showed reported benefit for IL-6 inhibitor therapy restricted to the anti-IL-6 receptor antibody preparation tocilizumab (WHO Rapid Evidence Appraisal for COVID-19 Therapies (REACT) Working Group, 2021). In contrast, no mortality benefit was observed in studies using the IL-6 blocker sarilumab, which has an identical biological mechanism of action as tocilizumab (antibody that binds IL-6 receptor) (WHO Rapid Evidence Appraisal for COVID-19 Therapies (REACT) Working Group, 2021). Reason two involves restriction of tocilizumab mortality benefit to studies employing weaker study design. Randomized controlled studies employing double-blinding and placebo were compared to open-label randomized controlled trials with neither double-blinding nor placebo (WHO Rapid Evidence Appraisal for COVID-19 Therapies (REACT) Working Group, 2021). Mortality benefit was restricted to open label unblinded studies without placebo. No mortality benefit was observed in studies employing double-blinding and placebo. A separate meta-analysis reported a similar pattern of findings with tocilizumab mortality benefit restricted to studies that did not include double-blinding or placebo (Tharmarajah et al., 2021). In fact, in this analysis all three tocilizumab studies with double-blinding and placebo showed nonsignificant increase in mortality in subjects given tocilizumab (Tharmarajah et al., 2021). We have previously expressed skepticism for rationale to use IL-6 inhibition to treat COVID-19 (Scherger et al., 2020). Therefore, absence of tocilizumab mortality benefit in trials with superior study design was anticipated, and we believe using this therapy in COVID-19 patients is dubious. Analysis of the mechanism of the discrepancy behind these two kinds of studies is presented below. Reason three is the underappreciated weight of evidence that must be ignored in order to accept a sepsis benefit due to inflammation-suppressing strategies like IL-6 blockade (Table 1). Benefit for tocilizumab to treat COVID-19 sepsis contradicts the history of failure of cytokine-inhibition therapy to treat sepsis in general. George Santayana reportedly said, “those who cannot remember the past are condemned to repeat it”.

## 6 COVID-19 and Corticosteroids; *Déjà Vu* all Over Again

The above quote attributed to the American baseball player Yogi Berra (1925–2015) refers to the tendency for history to repeat itself. Since COVID-19 is an example of sepsis (SARS-CoV-2 infection associated with advanced symptoms or signs, organ damage or death), hyperinflammation as the cause of disease was therefore implied (Lin et al., 2018). The Randomized Evaluation of COVID-19 Therapy (RECOVERY) trial concluded corticosteroid use in selected patients with COVID-19 lowered mortality, and dexamethasone treatment has been incorporated into COVID-19 treatment guidelines (RECOVERY Collaborative Group, 2021). This appears to represent a counterexample to failure of previous trials using corticosteroid therapy to lower sepsis mortality. There has been little discussion pointing out the striking outlier nature of the RECOVERY result. The established history of unsuccessful inflammation-suppressing treatments used for sepsis should urge caution in adopting this kind of COVID-19 therapy (Table 1). Two recent comprehensive reviews of corticosteroids in sepsis assessed 42 and 50 randomized controlled trials and reported no significant benefit in 28 days or short-term mortality (Rochwerg et al., 2018; Liang et al., 2021). Given historical precedent, we are dubious dexamethasone efficacy reported in RECOVERY indicates true corticosteroid efficacy in reducing COVID-19 mortality. Our concerns extend beyond historical steroid failure in sepsis. The ongoing RECOVERY trial uses an adaptive clinical trial design that employs randomization to one of several treatments and includes a usual or standard care arm as control. However, RECOVERY is neither blinded nor placebo controlled. Published assessments suggest these design omissions can bias trial results in favor of intervention (Roberts et al., 1993; Schulz et al., 1995; Hrobjartsson et al., 2012; Anthon et al., 2018; Saltaji et al., 2018). This concern is highlighted when examining a study similar to RECOVERY (Methylprednisolone as adjunctive therapy for patients hospitalized with Coronavirus Disease 2019 or Metcovid) that assessed the corticosteroid methylprednisolone for COVID-19 adjunct therapy (Jeronimo et al., 2021). Unlike RECOVERY, Metcovid employed double-blinding and placebo control. With inclusion of these safeguards against experimental bias, Metcovid did not demonstrate mortality benefit for corticosteroid use. Explanations that protect positive results in RECOVERY from discrepant mortality outcomes in Metcovid can be offered. Differences include use of alternative corticosteroid drugs (dexamethasone vs methylprednisolone), different patient populations and study designs, and smaller number of subjects in Metcovid. However, considering the remarkable history of failure of the hyperinflammation concept, we remain concerned the dexamethasone RECOVERY results are tenuous. Finally, we point out an historical precedent with striking parallels to RECOVERY dexamethasone results that raise additional cause for skepticism. A prior study by Fisher *et al* investigated the efficacy of adjunctive intravenous IL-1 receptor antagonist (IL-1ra) as a treatment for patients with severe sepsis or septic shock (Fisher et al., 1994b). This study enrolled 99 patients in a randomized, placebo controlled open label study that included three different doses of IL-1ra. Similar to RECOVERY, this trial was unblinded or open label, but unlike RECOVERY employed a placebo and explored a dose-response for IL-1ra. This study demonstrated significant mortality benefit using IL-1ra that also showed dose-responsive mortality reduction. However, two subsequent randomized, double blind, placebo controlled studies could not replicate the Fisher results and showed no mortality benefit for IL-1ra in sepsis (Fisher et al., 1994a; Opal et al., 1997). It is assumed the subsequent large phase 3 trials using strong experimental design revealed a true lack of benefit for IL-1ra in sepsis. This sequence of events is notable since the confirmatory studies employing double-blinding and placebo controls revealed the positive Fisher trial showing IL-1ra mortality benefit was incorrect. Although studying substantially fewer patients than RECOVERY, the positive Fisher study employed placebo control and conducted dose-response assessment of IL-1ra. These represent superior experimental design compared to RECOVERY. We see parallels between RECOVERY and the positive Fisher study and raise concern that subsequent definitive studies using superior methods will fail to replicate corticosteroid benefit in COVID-19. We point out the large RECOVERY trial also showed significant mortality benefit for tocilizumab in COVID-19. As discussed above, there are good reasons to question this conclusion as well.

## 7 Explaining Persistence of the Cytokine Storm Concept of Sepsis

**FIGURE 1:**
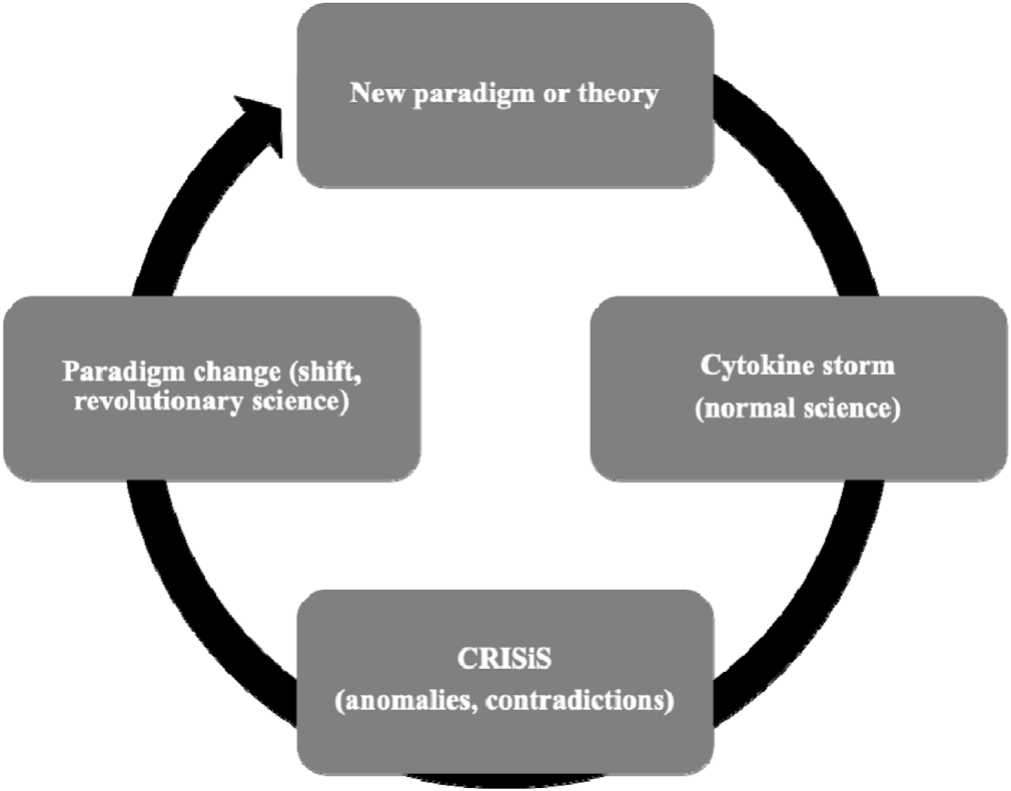
Adapted Kuhn cycle of scientific advancement using the cytokine storm paradigm as example.

Despite the troubled record of clinical applicability, the idea hyperinflammation is the cause of sepsis is rarely questioned. In fact, one may wonder if any amount of conflicting observation can disconfirm the cytokine storm concept. This pessimism is supported by sepsis reviews that acknowledge the weak track record of clinical progress in sepsis yet suggest future pathways that continue to follow the same hyperinflammation approach (Cross et al., 1993; Dinarello and Abraham, 2002; Vincent and Abraham, 2006; Rittirsch et al., 2007; Marshall, 2014; Tsirigotis et al., 2016; Cavaillon et al., 2020; De Stefano et al., 2020). Suggestions for future study run a bewildering gamut that includes blocking different cytokines or non-cytokine immune mediators, blocking inflammatory molecule combinations, suppressing inflammation at different times during the course of sepsis, inhibiting inflammation for different durations, altering doses of inflammation inhibitors, inventing biomarker instruments that identify optimal conditions of intervention, investing in genomic or proteomic research to determine the right targets, expand studies to target coagulation or complement, identifying special patient subsets that may benefit from anti-inflammation measures (Shakoory et al., 2016), bolstering inflammation by administration of pro-inflammatory mediators, devising novel sepsis diagnostic criteria, changing clinical endpoints like longer term mortality or disability, and altering design of clinical studies to more efficiently assess novel agents (Talisa et al., 2018). These suggestions prescribe altering almost everything except the hyperinflammation concept itself. We believe resistance of the hyperinflammation concept to contradicting results is striking and needs to be explained. A search for such an explanation led us to characterize the cytokine storm concept of sepsis as a scientific paradigm as conceived by Thomas Kuhn. The trajectory of scientific progress has been extensively studied by Thomas Kuhn (a physicist and philosopher of science) who popularized the terms “paradigm” and “paradigm shift” in his book titled: “The Structure of Scientific Revolutions” (Kuhn, 1962). Kuhn proposed science transitions through cycles that include discontinuous radical changes in scientific direction referred to as revolutionary science or paradigm shifts (Figure 1). Such shifts disrupt intervening periods of smooth accumulative scientific progress that he characterized as “normal” science or science conducted under direction of an existing paradigm. We believe applying Kuhn’s ideas about scientific progress provide the best available explanation for some peculiarities of the cytokine storm sepsis concept. If we cast the cytokine storm concept as the current paradigm directing investigation in a period of normal science, surprising persistence of the hyperinflammation approach becomes an anticipated phenomenon. We adapt features of Kuhn’s view of science to explain characteristics of the field of sepsis investigation that are otherwise puzzling. The two elements we find most interesting are the near-inexhaustible tolerance for repeated disconfirming clinical investigation (Table 1) and the lack of progress in enhancing patient care despite expenditure of massive resources to develop adjuvant immunomodulator sepsis therapies (conservatively estimated at >10 billion US dollars) (Marshall, 2014). Applying Kuhn’s view of scientific development to provide insight to understand the trajectory of sepsis investigation has been noticed previously. Artenstein *et al* invoke Kuhn’s thoughts on scientific progress to describe the course of anti-inflammation research in sepsis (Artenstein et al., 2013). Artenstein *et al* focus on supposed paradigm shifts in describing innovations in sepsis therapeutics (Artenstein et al., 2013). However, we believe the focus in Artenstein *et al* on Kuhn’s notion of paradigm shifts overlooks the most relevant contribution of Kuhn’s ideas to sepsis research. We believe characteristics of normal science or science conducted within an existing paradigm is most relevant for understanding peculiarities of the history of sepsis investigation.

### 7.1 Scientific Paradigm and Normal Science

The term “paradigm” is notoriously imprecise and detailed characterization exceeds the bounds of this report (Lakatos and Musgrave, 1970; Buchwald and Smith, 1997; Kindi and Arabatzis, 2012; Franklin, 2015; Orman, 2016). Kuhn’s view of paradigms evolved into the view that a paradigm is a disciplinary matrix (Kindi and Arabatzis, 2012). Due to extensive use and familiarity with the term “paradigm”, we use disciplinary matrix and paradigm interchangeably. We also select Kuhn concepts that readily apply to sepsis. A paradigm has two primary components that include scientific theory and exemplars. The theory component of a paradigm includes objects in nature (cytokines and other molecules, cells with various assigned functions, signaling molecules, receptors, drugs/therapies), defined patterns of interactions of the objects (innate immunity, inflammation, signaling pathways, cytokine storm, sepsis), and defined cause-effect relationships (inflammation causes organ malfunction or death, some molecules cause inflammation while other molecules suppress inflammation). Exemplars are concrete solved problems in science that serve as templates for teaching and research. They serve as guides for conduct of future investigation. They are paragons or especially clear solved problems that appear in textbooks and serve as precedents that are like legal precedent in case law. Effective exemplars demonstrate patterns of reasoning within a paradigm that demonstrate the pathway to future discoveries. A possible cytokine storm exemplar would be injection of endotoxin into animals with mortality reduction following administration of a TNF or IL-1 antagonist (Beutler et al., 1985; Ohlsson et al., 1990). A pivotal analogy formulated by Masterman compares paradigms to puzzle solutions (Lakatos and Musgrave, 1970). According to Masterman, normal science proceeds with the final experimental outcome known beforehand, but the pieces of the puzzle and how they fit together is specific to each of many experimental solutions to the puzzle. Given this analogy, we can characterize exemplars as especially transparent examples of how to solve puzzles and arrive at the correct answer repeatedly. By “correct” we refer to experimental results that support outcomes specified by the paradigm. According to Kuhn, the conduct of normal science is like puzzle solving and the goal of normal science is to solve as many interesting puzzles as possible. Importantly, the puzzle always looks the same, but the pieces keep changing shapes. The paradigm (final puzzle picture) is always the same even though the pieces used to make the puzzle can differ (different experiments). Applied to sepsis research, the puzzle pieces can differ according to different experimental designs, but the fnal picture defined by the pieces always shows cytokine storm. During the period of normal science, experimentation is conducted in service of an existing paradigm.

We consider current investigation in sepsis to be conducted under direction of the hyperinflammation or cytokine storm paradigm. Therefore, the field of sepsis research is operating within a period of normal sciences. Importantly, the paradigm defines which problems to pursue, what methodologies are used to pursue them, and what counts as a successful explanation for any problem under investigation (Kindi and Arabatzis, 2012). According to Kuhn, paradigms comprise the background conceptual scheme that directs the conduct and interpretation of research. The paradigm itself is never questioned. An interesting component of Kuhn’s concept of a paradigm is the evolution of a social structure including professional groups and institutions designed to project investigation into the future and includes funding organizations, professional journals and peer reviewers, guidelines for teaching, and standardized criteria for experimental methods. The social structure is designed to extend investigation into the future. It will also defend and augment the existing paradigm. Now let us apply Kuhn’s views on science to sepsis as an attempt to shed light on two peculiarities of sepsis study.

### 7.2 Life Within Normal Science: Paradigm Maintenance and Tolerance for Disconfirming Anomalies

In Kuhn’s view, normal science is highly resistant to anomalous or disconfirming observations, and data that do not fit an existing paradigm are ignored, altered in such a way as to be incorporated into the existing paradigm, or explained away by seeking reasons for experimental deviation from the existing paradigm. The paradigm itself is preserved at (nearly) all costs. A quote from Structure of Scientific revolutions is telling in this regard where Kuhn writes “it is a poor workman who blames his tools for a bad outcome” (Kuhn, 1962). Kuhn refers to pressure experienced by scientists to produce results that conform with the existing paradigm. Challenging the paradigm itself is not an option. Using the Masterman puzzle solving analogy of normal science, no matter what scientific question is posed in sepsis investigation, the answer is always hyperinflammation. The task for investigators is to construct an experimental path that solves the problem in a way that comports with hyperinflammation. As an example, consider research effort designed to understand COVID-19. Emergence of this disease presented many new challenges (puzzles) for investigators. Since current researchers operate within the hyperinflammation paradigm, these scientific puzzles were approached in a way that ensures cytokine storm is somehow characterized as the cause of disease. This accounts for the abundance of COVID-19 investigation that begins or concludes with elements of cytokine storm or hyperinflammation. It is no exaggeration to state reviews of COVID-19 pathogenesis always call forth the hyperinflammation paradigm.

Mechanisms that explain maintenance of the sepsis cytokine storm paradigm likely include two characteristics of paradigms. First, Kuhn’s emphasis on the role of a social structure that accompany paradigms is of special importance. Institutions enable and promote modern bioscience and they are heavily biased for care and maintenance of the current paradigm. Most scientists and physicians are educated to understand sepsis as a manifestation of cytokine storm and textbooks actively maintain the cytokine storm concept by depicting exemplars that encapsulate the contemporary approach to solving problems in infectious diseases. Journals favor manuscripts that offer experimental results that favor the hyperinflammation concept, and even lay press publications largely support the concept. Most importantly, funding agencies are populated with proponents of cytokine storm since adherence to this concept defines expertise in this field. A successful career in the modern era of bioscience requires large sums of funding to conduct research. Millions of US dollars are required to support even modest careers. Given these considerations, outlier scientists skeptical of cytokine storm face substantial difficulties obtaining resources with which to pursue such a controversial line of investigation. In fact, it is doubtful adherents to the current paradigm would view the work of anyone challenging that paradigm as conducting legitimate research. Funding agencies are unlikely to expend limited resources to support “speculative” experiments with a perceived small possibility of scientific return. Committing one’s career to being an outlier or “lone wolf” presents high risk for failure and such individuals are likely to receive little institutional, emotional, or financial support during career development. Second, conceptual confusion in the sepsis field is characterized by imprecision and weak definitions (Section 2). This can insulate the cytokine storm paradigm from falsification. Due to conceptual confusion, data of almost any sort can be interpreted as satisfying the paradigm. For example, clinical investigations often quantify multiple cytokines using multiplex assay platforms. Retrospective statistical analysis can usually generate association(s) between one or more cytokines and some clinical outcome or other. Since the concept of inflammation poorly characterizes which cytokines are pro-inflammatory, association between nearly every cytokine that can be measured with any of several clinical outcomes (mortality, use of oxygen, admission to intensive care unit, need for pressor support, etc) can be interpreted as a confirming instance of the cytokine storm sepsis paradigm. Imprecise sepsis-related concepts have led to retrospective re-examination of failed clinical trials to identify subgroups of patients that appeared to benefit from anti-inflammation interventions. Since concepts like inflammation or cytokine storm lack precision, these subgroups cannot be excluded as unlikely to benefit from anti-inflammation therapy. Examples of such subgroups include sepsis in a specific race (Bernard et al., 1997), bacterial etiology subgroups (Greenman et al., 1991; Ziegler et al., 1991; Fein et al., 1997), level of coagulopathy (Abraham et al., 2001; Abraham et al., 2003b), severity of sepsis at enrollment (Fisher et al., 1994a), and subgroups defined by circulating cytokine concentrations (Ferrara et al., 1993; Reinhart et al., 1996; Reinhart et al., 2001). No subgroup defined retrospectively led to successful therapy when subsequently studied prospectively. The cytokine storm paradigm is too imprecise to restrict expansion of subgroup analyses that serve up additional interventions based on cytokine storm.

Summarizing the points above, at least two characteristics of the cytokine storm paradigm foster persistence of this concept despite inability to deliver clinical treatment. First, social institutions formed around the cytokine storm paradigm direct the flow of many elements of science into this paradigm. A sepsis investigator encounters an educational history, funding agencies, and publishing organizations that channels the conduct of science to support the existing paradigm. Second, conceptual confusions in the sepsis field provide escape mechanisms that stymie falsification. Contradictory results are explained by failure of experimental design or conduct instead of paradigm failure. According to Kuhn, during normal science one possibility not to be considered is that investigation is conducted under direction of a faulty paradigm. Kuhn’s model of scientific progress appears to provide an understanding of how the current sepsis paradigm has survived despite apparent pressures to be discarded.

### 7.3 You Cannot Experiment Your Way out of a Failing Paradigm

Elements of theory within a paradigm establishes the menu of tools available for experimentation. This is a component of the theory-laden or theory-infection quality of experimentation (Franklin, 2015). Since we equate paradigm with a theory and associated exemplars, we refer to this idea as paradigm-laden or paradigm infection of experiment. As an example, sepsis experiments often use specific cell lines or animals, special stimuli like endotoxin or mitogens, selected culture conditions that specify atmospheric composition and temperature, outcomes that include cytokine quantification or other inflammation-related molecules. At every stage of experimentation from conception to design to available materials and protocols followed to interpretation of results, there is influence on experimental conduct exerted by cytokine storm orthodoxy. For a clear and striking example of how experiment is directed by paradigm or theory, consider the recent discovery of gravitational waves (Miller and Yunes, 2019). Existence of gravitational waves was inconceivable prior to the advent of Einstein’s theory of relativity since no prior paradigm contained a way to imagine gravitational waves. The relativity paradigm was used to design a unique experimental device called the Laser interferometer gravitation-wave observatory (LIGO) that did, in fact, detect gravitational waves in 2016. Note that LIGO was designed, engineered, and operated in accord with Einstein’s paradigm, demonstrating that a paradigm determines what is looked for in science and how one designs experiments that can find what you are looking for. Prior to Einstein, no amount of experimentation could possibly have discovered gravitational waves. Similarly, we are convinced experiments and clinical studies conducted within the cytokine storm paradigm are unlikely to lead to effective sepsis treatments. Decades of investment in experimentation under the guidance of cytokine storm has produced little therapeutic payoff. The pathway to advancement may not be through experimentation alone.

## 8 A Road Forward

If further experimentation is unlikely to provide meaningful advancement in sepsis therapeutics, we need to entertain alternative approaches. When applied to Kuhn’ model, the cytokine storm sepsis paradigm is in a state of normal science crisis. According to Kuhn, a scientific crisis is characterized by accumulation of anomalies that cannot be accommodated by an existing paradigm. Over time, accumulation of contradicting data can reach a point where the paradigm fails to function as a driver of scientific advancement and fails to solve puzzles. Normal science anomalies include contradictory observations (Table 1) and an inability to explain, predict, and control the empirical world. The criterion of control for cytokine storm focuses on delivering treatments based on suppressing inflammation. It is clear the cytokine storm paradigm cannot solve puzzles it should be able to solve. Figure 1 depicts a modified version of the Kuhn cycle of scientific progress.

### 8.1 Paradigm Shift

If we accept the verdict that the hyperinflammation sepsis paradigm is in crisis, resolution of the crisis will likely require a “paradigm shift.” Two impediments pose challenges for altering the current paradigm. The first impediment is Kuhn’s observation that the presence of crisis in science does not entail a search for paradigm alteration. In fact, shedding an existing paradigm is often the solution of last resort if it is considered at all. History shows anomalies or contradicting observations can be tolerated for prolonged periods of time and mechanisms are generated within the existing paradigm to explain anomalies (Kuhn, 1962).

As pointed out above, examination of commentaries acknowledging the presence of anomalies or failure of sepsis research to produce clinical benefit confirms this. The response to anomalies has been to invoke numerous suggestions for tweaking or rehabilitating the existing hyperinflammation sepsis paradigm. However, there has been no call to abandon the hyperinflammation or cytokine storm concept (Dinarello, 1991a; Cross et al., 1993; Vincent and Abraham, 2006; Rittirsch et al., 2007; Marshall, 2014; Tsirigotis et al., 2016; Cavaillon et al., 2020; De Stefano et al., 2020). This corresponds closely to Kuhn’s view that paradigm challenge is not an option that is entertained when things go wrong. The second impediment to sepsis paradigm change is pivotal. The Kuhn view includes the observation that an existing paradigm cannot be changed unless a viable alternative paradigm is presented as a replacement. Paradigms cannot be repealed until they are replaced, as stated explicitly in page 18 in Kindi and Arabatzis (Kindi and Arabatzis, 2012). Another way of stating this observation is to note paradigms are never falsified, they are replaced. Given the vital role a paradigm serves for conducting scientific research, it stands to reason an existing paradigm cannot be jettisoned without a replacement. In our view, lack of a replacement sepsis paradigm is the largest obstacle to generating sepsis therapies that will improve outcomes. Since it is unlikely a replacement paradigm can originate from experimentation, a replacement is more likely to emerge from investigators who function as medical theoreticians conversant with the basic research, clinical, and philosophical challenges associated with constructing a radically different view of sepsis. We are not aware of a sepsis paradigm alternative to cytokine storm in the public domain. However, our group has, in fact, devised a complete and consistent novel sepsis paradigm that has potential to replace cytokine storm. Discussing specific contents exceeds the focus of this report. However, some properties of this novel sepsis theory are noteworthy. It dispenses with any notion of hyperinflammation, explains why sepsis therapies have failed in the past, and proposes novel therapeutic approaches to treating sepsis.

## 9 Discussion

Standard of care for patients with sepsis includes rapid administration of antimicrobial drugs and supportive measures. However, despite advances in these areas, persistent substantial mortality is observed in sepsis. For this reason, attention has focused on adjunctive therapies targeting the pathogen-triggered host inflammatory response to infection. This approach follows a line of reasoning that posits hyperinflammation as the final common cause of organ malfunction or death. The COVID-19 pandemic has thrust hyperinflammation or cytokine storm to the forefront of discussion by scientific investigators, clinical trialists, and the lay public. Associating COVID-19 with cytokine storm was expected since severe COVID-19 is an example of sepsis, or infection sufficient to cause organ malfunction or death. Since sepsis is thought to be caused by hyperinflammation, a link between COVID-19 and hyperinflammation was forged. A substantial research tradition identifies pro-inflammatory cytokines as pivotal mediators of inflammation and are thus implicated as potentially reversible causes of host inflammation in response to infection. This accounts for use of the term “cytokine storm” to characterize sepsis hyperinflammation. Unfortunately, despite expenditure of ample resources to materially affect sepsis mortality by blocking inflammation, we have been unable to affect sepsis mortality using this concept. There is no indication we are on the verge of reversing this trend.

We believe the hyperinflammation or cytokine storm approach to sepsis is an imperiled concept. In this report we identify three elements that we believe are at the heart of difficulties with this concept. The three elements include 1) confusion and imprecision in descriptions of the hyperinflammation concept, 2) weak evidence supporting the idea hyperinflammation is the cause of sepsis, and 3) persistence of the hyperinflammation concept despite inability of therapies based on this concept to treat sepsis. First, we analyzed concepts that underly the hyperinflammation approach of sepsis. We found a surprising degree of conceptual confusion in pivotal ideas. The terms “inflammation” and “cytokine storm” are vague and imprecise. Considering inflammation, no explanation or definition precisely characterizes the systemic inflammation that presumedly causes sepsis. In fact, contemporary understanding of inflammation continues to refer to ideas present since antiquity. Prior sepsis definitions emphasized fever or hypothermia, tachypnea, tachycardia, and leukocytosis or leukopenia. How these patient data relate to inflammation is unclear. Inflammation manifests differently in separate individuals, and it can present in different ways in the same individual. An adequate account of inflammation must derive from an understanding of regularities present in all manifestations of inflammation. We believe such an account of inflammation must originate from a theory-driven characterization that directs selection of clinical or laboratory indicators that can identify and quantify inflammation. Relatedly, the term “cytokine storm” is vague and imprecise. We assume cytokine storm refers to excessive production of molecules that cause overexuberant inflammation and therefore sepsis. However, there is no consensus on which cytokines are relevant for production of inflammation. Moreover, there is no concept of what cytokine concentrations constitutes an excessive amount. Conceptual confusion has adverse scientific and clinical consequences. Given suppression of inflammation as a goal of sepsis therapy, we need to identify the right molecular targets to suppress. We believe progress in sepsis requires re-thinking concepts of inflammation and cytokine storm. Therefore, we have developed a novel conception of inflammation that attempts to demystify these concepts (manuscript in preparation).

Second, we analyzed the status of evidence supporting a cause-effect relationship between hyperinflammation and sepsis. Using criteria of necessity, sufficiency, and a pivotal interventionist criterion for causality, hyperinflammation or cytokine storm fails to satisfy criteria as a cause of sepsis.

Third, we have been struck by remarkable resilience of the hyperinflammation concept to contradicting clinical assessment. The concept persists with apparent indifference to a remarkable record of repeated inability to deliver clinical success (Table 1). It appears the concept is unfalsifiable by any amount of contradicting data. We believe Thomas Kuhn’s portrayal of scientific progress best explains this resilience. Kuhn’s description of the characteristics of normal science, or science conducted from within an established paradigm, is most applicable. It is reasonable to characterize hyperinflammation or cytokine storm as the current sepsis paradigm, where paradigm is the conceptual scheme that guides a scientific field. Paradigm components emphasize background theory and exemplars (Section 7). Defects we believe plague the cytokine storm paradigm include background knowledge (theory) that contains imprecise and poorly characterized concepts like inflammation and cytokine storm, mischaracterization of molecules/substances as pro- or anti-inflammatory, incorrect cause-effect relationships, and absent characterization of regularities relevant for an adequate theoretical description of sepsis. Exemplars are specific solved problems that serve as templates or patterns that guide future investigation. Objects and relations depicted in exemplars derive from theory and act as practical specific examples of how theory is supposed to apply to scientific observations. We fear currently employed exemplars are misguided and will direct research in unfruitful directions.

Kuhn refers to normal science as science conducted under the direction of a monpolistic paradigm. The paradigm is not questioned, and anomalous or contradictory observations are explained by errors of experimental design or experimental conduct. The underlying paradigm remains immune to disconfirmation and is insulated from experimental assessment. The Kuhn account of scientific progress anticipates problems that can originate from a faulty paradigm. The lack of precision or conceptual vagueness in the cytokine storm paradigm enables contradicting observations to be interpreted as consistent with hyperinflammation and cannot be used as falsifying examples. Failed clinical sepsis trails are nearly always viewed as failures of experimental design or conduct and are not used as reasons to challenge the underlying paradigm (Table 1). This accounts for suggestions to press on or “double down” by blocking different cytokines or combination blockade, treating different patient sub-populations, altering the dose or timing of anti-inflammation drugs, changing the outcome from mortality, and on and on ad infinitum. The one thing never questioned is the underlying hyperinflammation paradigm. A key implication is that paradigm change does not originate from experimentation alone. Indeed, the Kuhn concepts of scientific progress provides an account that explains resilience of the hyperinflammation or cytokine storm paradigm of sepsis despite substantial disconfirming data (Table 1). In accord with the Kuhn view of science, we believe a paradigm shift is long overdue in the field of sepsis. As Kuhn predicts, experimental triumph or failure is not the engine of paradigm change. The cytokine storm paradigm appears to represent a confirming example of Kuhn’s analysis of how science progresses. You cannot experiment your way out of a failing paradigm, and paradigms can be replaced but they cannot be falsified. We surmise the primary obstacle for paradigm shift in sepsis is unavailability of an alternative paradigm. Existing paradigms are never abandoned in the absence of a novel paradigm ready for adoption. Our group has developed such a novel sepsis paradigm that invokes no role for inflammation whatever. This will be the subject of a separate report. Finally, the ghost mentioned in our title refers to use of hyperinflammation or cytokine storm to understand and treat sepsis. We believe this paradigm is based on an illusion related to a reversal of cause-and-effect. Continued pursuit will be as unproductive as ghost hunting. We believe inflammation and sepsis are not causally related and inflammation has little, if anything, to do with sepsis. We have been chasing the nonexistent ghost of hyperinflammation in the belief it will somehow explain sepsis or provide guidance to improve patient care.

## Data Availability

The original contributions presented in the study are included in the article/Supplementary Material, further inquiries can be directed to the corresponding author.

## Abbreviations

COVID, Coronavirus disease 2019; CTAP, Coronavirus treatment acceleration program; FDA, food and drug administration (United States); IL, interleukin; IL-1ra, IL-1 receptor antagonist; Metcovid, methylprednisolone as adjunctive therapy for patients hospitalized with Coronavirus Disease 2019; RECOVERY, randomized evaluation of COVID-19 therapy; SARS-CoV-2, severe acute respiratory syndrome Coronavirus 2; SCCM, society of critical care medicine; SIRS, systemic inflammatory response syndrome; SOFA, sequential organ failure assessment; TNF, tumor necrosis factor alpha.
